# Effect of Mobile Augmented Reality Counseling on Improving Shared Decision-Making in Thoracic Surgery: Randomized Clinical Crossover Trial

**DOI:** 10.2196/79632

**Published:** 2025-11-13

**Authors:** Ying-Shian Chen, Yi-Chen Hsu, Worachate Romalee, Ding-Han Wang, Jennifer Lai, Tsai-Wang Huang, Kuan Hsun Lin

**Affiliations:** 1 School of Medicine National Defense Medical University Taipei Taiwan; 2 Taichung Armed Forces General Hospital Taichung Taiwan; 3 Division of Thoracic Surgery, Department of Surgery, Tri-Service General Hospital National Defense Medical University Taipei Taiwan; 4 College of Dentistry National Yang Ming Chiao Tung University Taipei Taiwan; 5 Oral Medicine Innovation Center (OMIC) National Yang Ming Chiao Tung University Taipei Taiwan; 6 Department of Community Dentistry & Gerodontology, Faculty of Dentistry Thammasat University Pathum Thani Thailand; 7 Research Center for Intelligent Sensor Device National Yang Ming Chiao Tung University Taipei Taiwan; 8 Tri-Service General Hospital National Defense Medical University Taipei Taiwan

**Keywords:** mobile augmented reality, shared decision-making, patient education, thoracic surgery, 3D models

## Abstract

**Background:**

Augmented reality (AR) superimposes virtual objects onto a real-world environment, allowing users to interact in real time. As AR has become widely used, its integration into smartphones or tablets has enabled mobile augmented reality (MAR) experiences. AR has been adopted in many industries, and the literature has highlighted its applications in academic and clinical settings, particularly in enhancing visualization, communication, and learning.

**Objective:**

This study investigated the potential of MAR as a mobile health tool to enhance shared decision-making (SDM) in thoracic surgery by increasing patient understanding and engagement during medical consultations.

**Methods:**

A randomized crossover clinical trial was conducted at the Tri-Service General Hospital in Taiwan. Participants scheduled for thoracic surgery were enrolled and randomized in a crossover design. The MAR intervention incorporated patient-specific 3D anatomical models that were reconstructed from computed tomography imaging to facilitate understanding and support SDM. The impact of each counseling approach on SDM was evaluated using postintervention questionnaires.

**Results:**

A total of 47 participants were enrolled in this study. After analyzing the data, we found that patients in the MAR group showed significantly higher scores compared to those in the traditional counseling group (*P*<.001) during the SDM process. Moreover, patients reported higher satisfaction levels and found the visual objects helpful for understanding tumor location and surgical procedures.

**Conclusions:**

This study demonstrated that MAR counseling significantly enhanced patients’ comprehension of thoracic conditions and increased their active engagement in the SDM process (*P*<.001). The integration of patient-specific 3D anatomical models into MAR technology provided an intuitive method for critical medical information. This digital approach not only enhanced personalization in medical communication but also reinforced patient education about their health care conditions. These findings suggest that MAR counseling represents a promising approach for promoting patient-centered care in thoracic surgery and has potential applications across various clinical domains.

**Trial Registration:**

ClinicalTrials.gov NCT07062393; https://clinicaltrials.gov/study/NCT07062393

## Introduction

### Role of Mobile Augmented Reality in Clinical Counseling and Mobile Health

Augmented reality (AR) allows the seamless integration of virtual elements with real-world environments, including the superimposition of digital files, such as 3D models, animations, and videos. This enhances interactive learning, visualization, and communication in medical contexts [1]. A more accessible and scalable evolution of AR is mobile AR (MAR), which operates via smartphones and tablets [2]. Unlike AR headsets or smart glasses, MAR leverages everyday mobile devices to deliver immersive experiences, making it a cost-effective and broadly deployable solution in both clinical and remote settings.

In the medical field, AR has been increasingly applied in thoracic surgery for training, preoperative planning, and intraoperative assistance [3]. However, a noticeable gap exists in the literature regarding the use of MAR as a patient-facing mobile health (mHealth) tool to improve communication and support shared decision-making (SDM). By enabling real-time 3D visualization of patient-specific anatomy through mobile devices, MAR represents a novel strategy within the mHealth paradigm to promote accessibility, patient engagement, and autonomy in surgical counseling.

### Surgical Counseling Challenges in Thoracic Surgery

Lung cancer is a global health challenge, as reflected in the 2020 world statistics. The incidence of lung and bronchus cancer was reported at 31.5 cases per 100,000 population for men and 14.6 cases per 100,000 population for women. The widespread prevalence of lung cancer continues to affect a significant portion of the global population [4].

In Taiwan, the situation is even more concerning. According to the latest report by Taiwan’s Ministry of Health and Welfare in 2021, the rate of new cases of lung and bronchus cancer was alarmingly high, at 77.39 per 100,000 population for men and 67.13 per 100,000 population for women. Among the 16,880 newly diagnosed cases, a considerable portion (40.4%) received surgical treatment [5], which is one of the essential treatment options and the standard approach for early-stage disease.

The surgical approaches for lung resection could be divided into open thoracotomy and minimally invasive thoracic surgery (ie, video-assisted and robot-assisted thoracoscopic surgery). The various modes of lung resection, including anatomical (pneumonectomy, lobectomy, and segmentectomy) and nonanatomical (wedge resection) [6], pose a significant challenge to patient understanding. Traditional methods of patient counseling, relying on oral explanations, 2D computed tomography scans, and schematic diagrams, often fail to provide a comprehensive understanding. Although informative, these methods do not fully convey the intricacies of each surgical option, leaving patients inadequately equipped to make informed decisions.

This gap in understanding is critical, as patients need to comprehend various aspects of their condition, such as tumor location, its proximity to vital structures, and the specifics of different surgical procedures, each with its unique risks and implications. The limitations of current counseling methods highlight the necessity for innovative approaches that can enhance physician-patient communication, provide better visual support, and enable patients to engage more effectively in their treatment planning.

### Communication and SDM

Several factors contribute to the quality of patient counseling and decision-making. These include patient anxiety, cultural background, insufficient medical knowledge, and awareness, all of which can significantly influence clinical decision outcomes [7,8]. Patients tend to participate more actively in treatment planning when they clearly understand their medical information [9]. Ineffective communication, especially when lacking meaningful patient engagement [10], may result in misunderstandings, dissatisfaction, disputes, and even violence against health care providers [11]. SDM has been increasingly promoted to emphasize patient preferences, values, and autonomy in the medical decision-making process [12]. It is now widely endorsed by academic and professional societies as a means to improve decision quality [13]. In addition, assistive tools—especially visual and interactive aids—have also been shown to support this process [14].

### Study Objective

To address this unmet need, we developed a MAR-based counseling system that integrates patient-specific 3D visualization into the SDM process for thoracic surgery. This study introduces a novel application of mHealth aimed at bridging the gap between complex surgical information and patient understanding.

## Methods

### Study Design

This study was a single-center, 2-arm, randomized crossover clinical trial conducted at Tri-Service General Hospital, Taipei, Taiwan, between July 2022 and June 2023. The study compared traditional image-based counseling with MAR-enhanced counseling for patients undergoing thoracic surgery. Participants were randomized to 1 of 2 counseling sequences: traditional followed by MAR (group A) or MAR followed by traditional (group B). Each participant received both formats of counseling during a single encounter. To evaluate the impact of each method, participants completed self-administered questionnaires after each session to assess SDM and counseling preferences. No formal washout period was implemented due to the educational and noninterventional nature of the study.

### Patient and Public Involvement

Patients or the public were not involved in the design, conduct, reporting, or dissemination of this study, as the research focused on the technical development and initial validation of a mobile-based counseling tool. Future iterations may incorporate patient feedback to optimize usability and relevance.

### Sample Size Calculation

Sample size was calculated using G*Power (version 3.1.9.7; Heinrich Heine University Düsseldorf) to detect a significant difference between 2 independent groups using nonparametric analysis. The calculation was based on unpublished pilot data from our research team, involving 10 patients who received both MAR and traditional counseling. A large effect size (Cohen *d*=0.9) was observed in SDM score differences. Based on a two-tailed test with an alpha level of .05 and statistical power of 0.80, the required sample size was calculated to be 44 participants (22 per group). To accommodate for potential dropouts, 47 patients were ultimately enrolled.

### Recruitment

Participants were recruited through outpatient and inpatient referrals from thoracic surgery clinics. Inclusion criteria were as follows: (1) scheduled for thoracic surgery (eg, lobectomy, segmentectomy, or wedge resection); (2) aged 20 to 65 years; (3) capable of using a smart device; (4) able to communicate in Mandarin or Taiwanese; (5) willing and able to provide informed consent; and (6) capable of completing questionnaires independently or with minimal assistance. Exclusion criteria were as follows: (1) emergency surgery, (2) severe cognitive impairment or psychiatric illness interfering with participation, (3) visual or hearing impairments interfering with mobile device interaction, (4) prior participation in similar counseling studies, and (5) inability to complete follow-up.

### Randomization

Consenting participants were randomly allocated to either group A (traditional counseling first) or group B (MAR counseling first) using a basic random number generator by a research assistant without allocation concealment or stratification. Due to the nature of the interventions, blinding was not feasible in this study.

### Interventions

A standardized counseling protocol was developed to ensure consistency across both groups and was delivered by the same thoracic surgeon to minimize interoperator variability. During crossover sessions, surgeons maintained neutrality and avoided providing additional interpretation. Surgeons adhered to the protocol and followed a structured sequence to explain the tumor location, surgical options, and associated risks.

In group A, participants received traditional counseling first, which involved an explanation of their health condition using verbal explanations supported by axial, coronal, and sagittal CT images and a 10 to 15-minute discussion. They were asked to complete a questionnaire related to SDM (questionnaire 1), followed by MAR counseling with interactive, patient-specific 3D anatomical models presented through mobile devices, and then to fill out a second questionnaire (questionnaire 2). In contrast, participants in group B began with MAR counseling. This part included a 5-minute app introduction followed by a 10-to-15-minute personalized anatomical visualization and discussion using the MAR application. After participants (specifically to participants who are in group B) completed the questionnaire 1, they received traditional counseling and proceeded to complete questionnaire 2. All participants experienced both counseling approaches, and their overall preferences and perceptions were evaluated through a questionnaire 3. In Multimedia Appendix 1, demonstration of both traditional and mobile augmented reality (MAR) counseling methods is shown in MP4 format.

### Patient-Specific 3D Models via MAR Application

**Figure 1:**
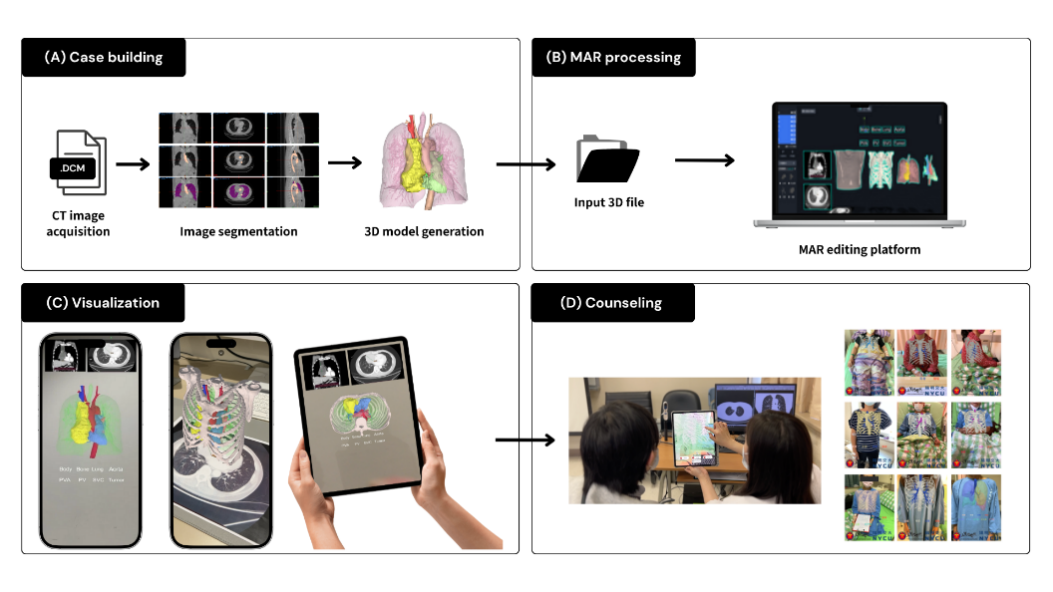
Preparation workflow for building a mobile augmented reality (MAR) system. (A) Acquiring patient-specific computer tomography (CT) data, performing anatomical segmentation, and generating models in a 3D format. (B) Models are optimized and uploaded to the editing platform to adjust settings. (C) Models are embedded into the application’s interface, enabling interactive manipulation (zoom, rotation). (D) The finalized interactive model is deployed to the mobile device for physician-patient counseling.

CT images of 47 patients scheduled for thoracic surgery at the Tri-Service General Hospital were collected. Digital images in Digital Imaging and Communications in Medicine format were acquired and imported into Materialise Mimics software (version 22; Materialise NV) for anatomical segmentation and 3D reconstruction of the lung and tumor regions (Figure 1A). Subsequently, we meticulously crafted standard tessellation language 3D models and transformed these Standard Tessellation Language models into GLB format models in Blender software (version 3.6, Blender Foundation) to ensure accurate color representation. The finalized GLB files were then uploaded into the MAR Editor platform (version 3.4.2, Mifly). In the process of editing, we enhanced the visualization by assigning realistic colors, transparency settings, and interactive marks (Figure 1B). The editing platform allowed real-time interaction with the model on tablets or smartphones. Each model was fully interactive, allowing rotation, zooming, and exploration of the anatomical structures from multiple angles (Figure 1C). During counseling sessions, surgeons used these personalized models to explain the disease, treatment options, and the anticipated surgical procedures. (Figure 1D). For standardization, all participants interacted with the same mobile device, an iPad Pro (12.9 inch; fourth generation; Apple Inc), during MAR counseling sessions. In Multimedia Appendix 2, step-by-step demonstration of the mobile augmented reality (MAR) 3D model creation and deployment is shown in MP4 format.

### Measurement of Satisfaction Outcomes

To assess the impact of the different counseling methods on SDM and user preference, we designed the self-reported questionnaires for this study. Questionnaire 1 focused on SDM outcomes following the counseling session and included 10 items. Questionnaire 2 was the same as questionnaire 1, allowing the acquired preferences to be determined by comparing the results of questionnaire 2 with those of questionnaire 1. Questionnaire 3 specifically evaluated participants’ overall preference for a traditional or an MAR counseling approach. All questionnaire items were rated on a 5-point Likert-type scale, ranging from 1 (“strongly disagree”) to 5 (“strongly agree”). A higher score indicated a stronger impact of patient counseling on SDM. To ensure content validity, the questionnaires were reviewed and refined with input from an expert panel.

The primary outcome was the level of SDM assessed in each counseling session (questionnaire 1 and questionnaire 2). The secondary outcome was participant preference (questionnaire 3).

### Data Collection and Statistical Analysis

All 47 participants completed the questionnaires, and no one was excluded from the analysis. After the questionnaires were collected and anonymized in Microsoft Excel, relevant statistical analyses were performed using SPSS (version 24.0; IBM Corp). Descriptive statistics were used to summarize the baseline characteristics, demographic data, and outcome variables. Group differences were assessed using Fisher exact and the chi-square tests for categorical variables. To evaluate group comparisons and relationships among variables, independent 2-tailed *t* tests, paired 2-tailed *t* tests, Pearson correlation analyses, and multiple linear regression models were conducted. We recognized that in a crossover study, participants receiving the experimental variable in the first period might continue to be affected in the second period. To minimize carryover effects, we applied a linear mixed model analysis, considering sequence and period effects to adjust the influencing factors. *P*<.05 was considered statistically significant.

Given the noninterventional and educational nature of this study, no interim analyses or stopping guidelines were planned.

### Ethical Considerations

The study was reviewed and approved by the institutional review board of Tri-Service General Hospital (A202205082) and was retrospectively registered on ClinicalTrials.gov (NCT07062393). Written informed consent was obtained from all participants before enrollment after being informed of the study purpose, procedures, and voluntary nature of participation. The study posed minimal risk, involved no invasive procedures, and used patients’ own clinical data for educational counseling purposes. No additional imaging was required. No concomitant care or cointerventions were provided during the trial period. Participation was voluntary, with the right to withdraw at any time. All data were securely stored and anonymized before the analysis to ensure participant confidentiality. Access to identifiable information was restricted to authorized research personnel. No financial or material incentives were provided to participants.

## Results

### Participant Characteristics

**Figure 2:**
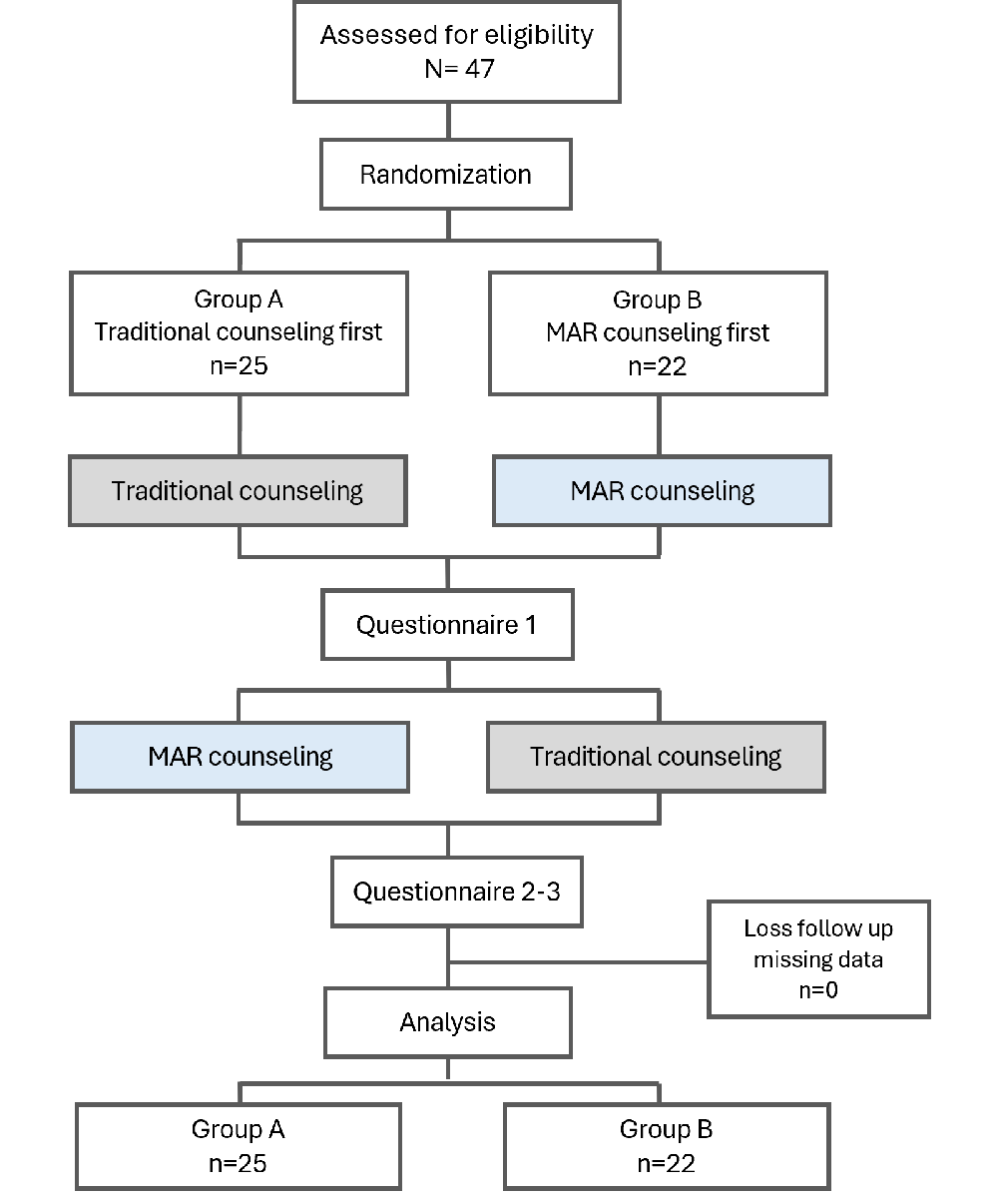
Study flowchart for participant enrollment, randomization, intervention, and analysis. MAR: mobile augmented reality.

A total of 47 participants fully completed questionnaires and were included in the final analysis, with 25 from counseling group A and 22 from counseling group B. A visual summary of the study flow is provided in Figure 2. No adverse events or unintended effects were reported in either group during or after the counseling sessions. The interventions involved no physical or psychological risks, and all participants completed the study without incident. Most participants in both groups were older than 30 years, held a university degree or higher, had been using smartphones for 10 to 20 years, and had no experience with AR or virtual reality (VR) technologies. No significant differences were observed in participant characteristics between the two groups, as summarized in Table 1.

**Table 1:** Demographic characteristics of participants (N=47).

Characteristics			Group A (n=25)		Group B (n=22)		*P* value	
**Sex** **n (%)**								.12^a^
	Male	8 (32%)		12 (54.6)				
	Female	17 (68)		10 (45.4)				
**Age (years), n (%)**								.20^b^
	<30	1 (4)		2 (9.1)				
	30-59	12 (48)		15 (68.2)				
	≥60	12 (48)		5 (22.7)				
**Education, n (%)**								.35^a^
	High school or lower	7 (28)		9 (40.9)				
	University or higher	18 (72)		13 (59.1)				
**Duration of mobile phone use (years), n (%)**								>.99^b^
	≤10	6 (24)		5 (22.7)				
	10-20	16 (64)		15 (68.2)				
	≥20	3 (12)		2 (9.1)				
**Average daily internet use (hours), n (%)**								.64^a^
	<3	10 (40)		7 (31.8)				
	3-5	10 (40)		8 (36.4)				
	≥5	5 (20)		7 (31.8)				
**Experience of using health care application, n (%)**								.91^b^
	Every day	7 (28)		7 (31.8)				
	Every week	5 (20)		4 (18.2)				
	Every month	3 (12)		4 (18.2)				
	Less than once a month	4 (16)		4 (18.2)				
	Never	6 (24)		3 (13.6)				
**Experience of using AR^c^ and VR^d^, n (%)**								.20^b^
	Yes	4 (16)		7 (31.8)				
	No	21 (84)		15 (68.2)				

^a^Chi-square test.

^b^Fisher exact test.

^c^AR: augmented reality.

^d^VR: virtual reality.

### SDM Scores

Table 2 presents the results of the questionnaire related to SDM. Before grouping, the overall mean score for traditional counseling was 37.40 (SD 5.83), and the overall mean score for MAR counseling was 47.98 (SD 2.92). The results related to patient SDM scores showed a significant difference (*P*≤.001). This finding indicates that MAR counseling was more effective in supporting patients’ engagement in the decision-making process. In traditional counseling, the lowest-scoring topics were encouraging participation (mean 3.66, SD 0.70) and enhancing communication (mean 3.66, SD 0.67). In contrast, MAR counseling achieved significantly higher scores in these 2 topics (mean 4.83, SD 0.38 and mean 4.87, SD 0.34; *P*<.001), including thought organization (mean 4.83, SD 0.38) and trusting health care providers’ treatment methods (mean 4.83, SD 0.38). MAR counseling also supported participants in understanding medical options (mean 4.74, SD 0.44) and provided more opportunities to ask questions to health care providers (mean 4.77, SD 0.43), thereby supporting decision-making (mean 4.79, SD 0.41).

**Table 2:** Results from the 10-item questionnaire assessing patients’ perspectives on shared decision-making (SDM) after traditional versus mobile augmented reality (MAR) counseling.

Questions	Traditional counseling, mean (SD)	MAR counseling, mean (SD)	*P* value^a^
1. Helps me feel more prepared to make decisions.	3.81(0.71)	4.79 (0.41)	≤.001
2. Helps me organize my thoughts about these decisions.	3.83 (0.64)	4.83 (0.38)	≤.001
3. Helps me consider the pros and cons of each option.	3.74 (0.68)	4.77 (0.48)	≤.001
4. Helps me compile the questions I want to ask.	3.69 (0.67)	4.77 (0.43)	≤.001
5. Clarifies and communicates what matters most to me.	3.72 (0.62)	4.79 (0.41)	≤.001
6. Helps me understand the available medical options.	3.81 (0.68)	4.74 (0.44)	≤.001
7. Provides me with the opportunity to ask questions.	3.68 (0.63)	4.77 (0.43)	≤.001
8. Encourages my participation in the health care process.	3.66 (0.70)	4.83 (0.38)	≤.001
9. Enhances communication between me and health care providers	3.66 (0.67)	4.87 (0.34)	≤.001
10. Helps me trust health care providers’ treatment methods	3.77 (0.73)	4.83 (0.38)	≤.001

^a^*P* values were obtained using the Wilcoxon rank-sum test. Higher scores indicate greater agreement with each statement (1-5 Likert scale).

**Figure 3:**
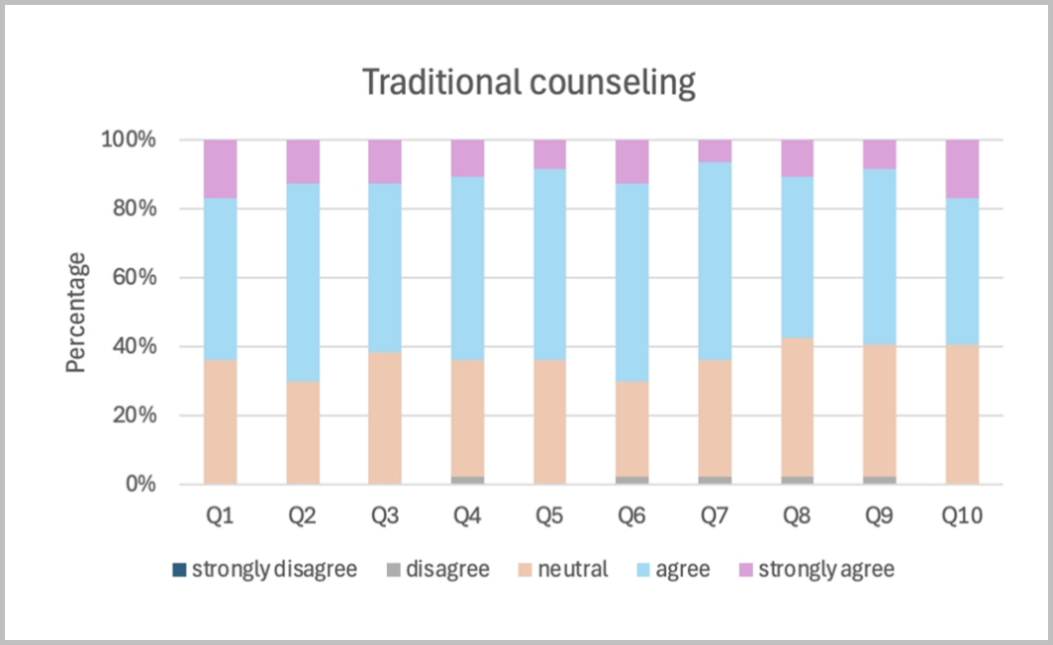
Distribution of participant responses to each item of the 10-item Shared Decision-Making Questionnaire (SDM-Q-10) following traditional counseling. Responses were measured on a 5-point Likert scale ranging from "strongly disagree" to "strongly agree." Item wording is provided in Table 2.

**Figure 4:**
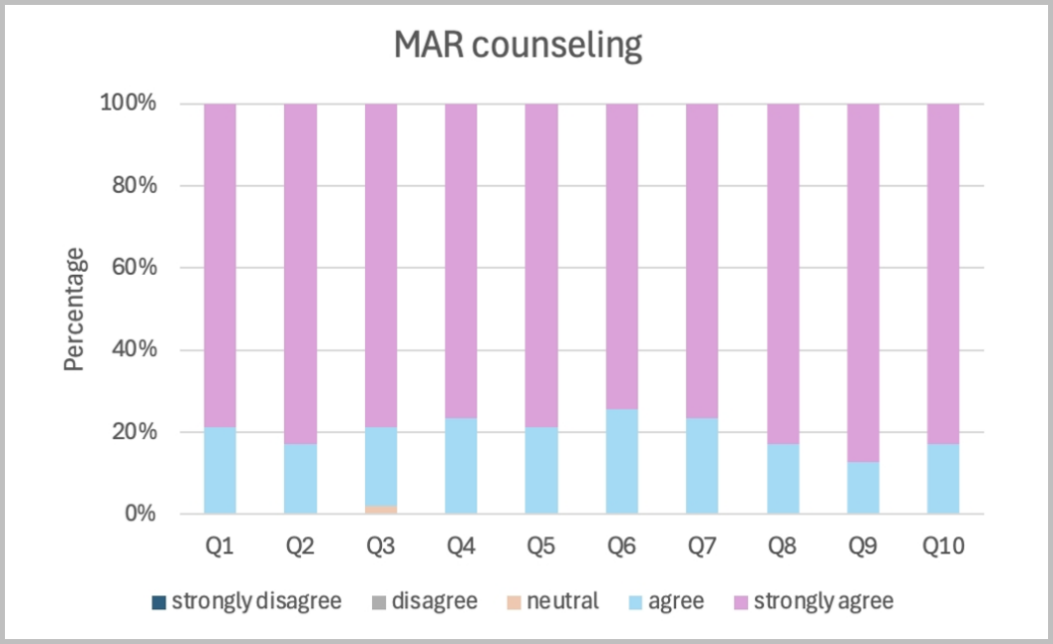
Distribution of participant responses to each item of the 10-item Shared Decision-Making Questionnaire (SDM-Q-10) following mobile augmented reality (MAR) counseling. Responses were measured on a 5-point Likert scale ranging from "strongly disagree" to "strongly agree." Item wording is provided in Table 2.

Figures 3 and 4 illustrate the distribution of participant responses for each of the 10 questionnaire items following traditional and MAR counseling. In traditional counseling, most participants rated between “agree” and “neutral,” with a few selecting “strongly agree.” By contrast, the MAR counseling demonstrated higher agreement across all topics, with nearly all participants selecting “agree or strongly agree.” The lowest-scoring item, considering the pros and cons of each treatment option (question 3), was rated at 95.46%. For all other topics, 100% of participants rated either agree or strongly agree, with virtually no neutral or negative responses.

### Interventions in Crossover Design

To evaluate the effect of counseling sequence on participant satisfaction, an independent sample *t* test was conducted to compare the differences between group A and group B in this crossover design. Participants were randomly assigned to either group A, which received traditional counseling followed by MAR counseling, or group B, which received MAR counseling first, followed by traditional counseling. The results showed no significant differences between the 2 sequences. The mean satisfaction score for traditional counseling was 36.92 (SD 6.07) in group A and 37.95 (SD 5.64) in group B (*t*=−0.60, *P*=.55), whereas MAR counseling scored 47.72 (SD 2.94) and 48.27 (SD 2.95) in group A and B, respectively (*t*=−0.64, *P*=.52) These findings indicated that the order in which traditional and MAR counseling methods were administered did not influence satisfaction outcomes.

Further within-group analyses revealed that MAR counseling was consistently rated higher than traditional counseling across both sequences. In group A, the mean difference between traditional and MAR counseling was −10.80 (SD 5.65, *P*<.001, Cohen *d*=−1.91), and in group B, the difference between MAR and traditional counseling was 10.32 (SD 5.61, *P*<.001, Cohen *d*=1.84).

### Adjusted Comparison Using Linear Mixed Model

**Figure 5:**
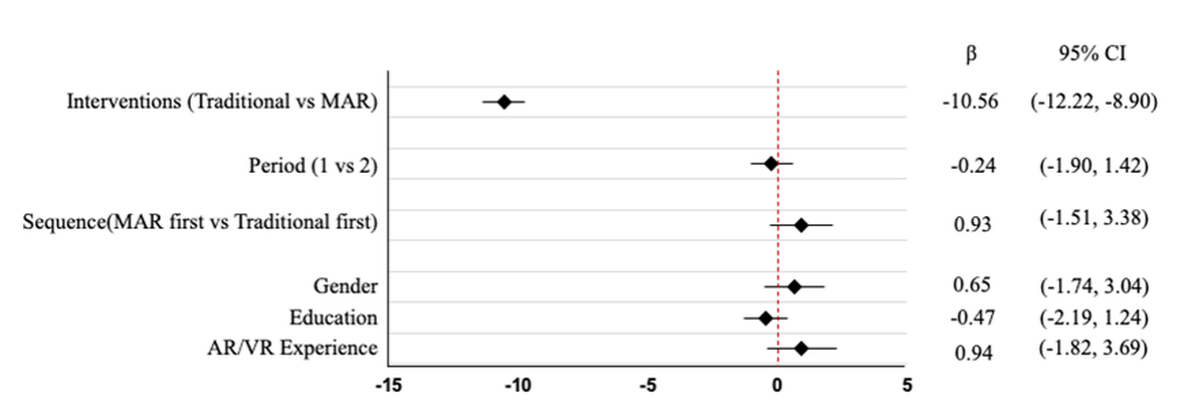
Fixed effects estimated from linear mixed model assessing the impact of treatment sequence, period, and demographic variables on shared decision-making (SDM) scores. AR: augmented reality; VR: virtual reality; MAR: mobile augmented reality.

Although the paired samples *t* test provided preliminary evidence that MAR counseling was rated higher than traditional counseling, additional analyses using a linear mixed model were conducted to examine any potential carryover effect. Considering both the sequence (traditional first or MAR first) and the period (first or second), neither the period nor the sequence effect reached statistical significance (*P*>.05). Demographic variables, including gender, education level, and AR and VR experience, also did not significantly affect the outcomes. However, the analysis showed that the different interventions demonstrated a significant difference; MAR counseling scored 10.55 points higher than traditional counseling, reaching statistical significance (*P*≤.001). These findings indicate that the type of intervention was the primary factor influencing participants’ perceptions; whereas period and sequence effects did not significantly impact counseling outcomes. The fixed-effect estimates are presented in Figure 5.

### Participant Preferences

The participants’ overall preferences were assessed with questionnaire 3 (I am willing to use this counseling method to communicate with health care providers; 1=traditional; 2=MAR). The analysis revealed that participants had a strong preference for the MAR counseling method. The mean score for MAR counseling (4.96, SD 0.20) was significantly higher than that for traditional counseling (mean 3.77, SD 0.70; *P*≤.001). Furthermore, 45 of 47 (96%) participants indicated a strong willingness to use MAR counseling as a communication method in the SDM process.

### Influence of Demographic Variables

To further investigate whether the participants’ preferences were influenced by demographic variables, a multiple linear regression analysis was conducted (Table 3). The results showed that none of the demographic variables, including gender, education level, duration of mobile phone use, average daily internet use, and AR and VR experience, reached statistical significance (*P*>.05) in the MAR counseling group. Similarly, most of the demographic variables did not demonstrate a significant result in traditional counseling. However, without AR and VR experience showed a positive effect (B=0.583, β=.357; *P*=.03), indicating that the participants without AR and VR experience reported a higher preference for the traditional counseling approaches compared to those who had the experience in AR and VR technology. The adjusted *R*^2^ values for both models were low (MAR=0.12; traditional=0.19), indicating that demographic factors explained a small proportion of the results.

**Table 3:** Multiple linear regression predicting shared decision-making scores from demographic variables.

Variable			B (95% CI)		β		*t* test (*df*)		*P* value
**MAR^a^ counseling**									
	Sex (female)	0.092 (−0.034 to 0.217)		.224		1.476 (40)		.15	
	Education level (college)	−0.037 (−0.185 to 0.110)		−.092		−0.51 (40)		.61	
	Education level (graduate)	−0.147 (−0.341 to 0.047)		−.259		−1.532 (40)		.13	
	Duration of mobile phone use	−0.003 (−0.121 to 0.116)		−.008		−0.048 (40)		.96	
	Average daily internet use	0.026 (−0.056 to 0.109)		.106		0.641 (40)		.52	
	AR^b^ and VR^c^ experience (no)	−0.013 (−0.173 to 0.148)		−.027		−0.161 (40)		.87	
**Traditional counseling**									
	Sex (female)	0.198 (−0.215 to 0.611)		.142		0.969 (40)		.34	
	Education level (college)	0.253 (−0.232 to 0.738)		.183		1.054 (40)		.30	
	Education level (graduate)	0.517 (−0.121 to 1.155)		.266		1.637 (40)		.11	
	Duration of mobile phone use	−0.108 (−0.498 to 0.282)		−.089		−0.561 (40)		.58	
	Average daily internet use	0.045 (−0.228 to 0.317)		.053		0.332 (40)		.74	
	AR and VR experience (no)	0.583 (0.055 to 1.111)		.357		2.232 (40)		.03	

^a^MAR: mobile augmented reality.

^b^AR: augmented reality.

^c^VR: virtual reality.

## Discussion

### Principal Findings

The study results indicated that participants preferred MAR counseling more than traditional counseling approaches during SDM, particularly regarding trust and participant engagement. This effect may be attributed to participants gaining more disease-specific knowledge from MAR, which, in turn, led to increased engagement [9]. Paired samples *t* tests revealed significantly higher scores for MAR counseling, demonstrating that the digital approach had a positive impact in helping participants comprehend the risks and benefits associated with their treatment options. Furthermore, the findings were strengthened by the linear mixed model analysis, which considered sequence and period effects to adjust the impact factors. Neither the sequence nor the period effects reached statistical significance.

These findings are consistent with those of a previous study that used 3D models in patient counseling for renal masses [15]. Although it is possible that the visually rich nature of MAR contributed to patient engagement, the structured questionnaires assessed more than just visual appeal. Notably, items such as question 3 (“Helps me consider the pros and cons of each option”) and question 6 (“Helps me understand the available medical options”), which evaluated the understanding of pros and cons and treatment options, showed significant improvement in the MAR counseling. This suggests that MAR facilitated greater engagement and deeper cognitive processing of medical information. These findings indicate that the observed improvements in SDM were not limited to increased engagement or visual novelty but included measurable enhancement in understanding treatment risks and options. This supports the value of MAR not only as a communication aid but also as a decision-support tool grounded in cognitive comprehension that strengthens SDM processes in clinical practice.

### Implementation Considerations

However, several considerations should be noted. MAR counseling demanded more time and a more complex preparation process compared with traditional counseling. The cost-effectiveness of adopting this new counseling method should be explored thoroughly before full implementation.

MAR offers a powerful solution to enhance telemedicine and improve technological literacy. One key advantage is its ability to address the challenge of interpreting complex medical data, such as 2D DICOM images, which can be difficult for patients without specialized anatomical knowledge to understand. MAR overlays 3D visualizations onto the physical world, making it easier for patients and health care providers to comprehend medical images by offering an interactive, spatial representation of anatomy. This interactive experience enhances the clarity of diagnostic information, allowing for more informed SDM.

Moreover, unlike the use of AR smart glasses, which can sometimes cause discomfort such as dizziness or visual fatigue, patients using MAR on mobile devices did not report such issues. The display on a mobile device offers flexibility in positioning and control, reduces strain on the eyes, and prevents the sensory overload that can occur with head-mounted displays. As a result, patients were more comfortable engaging with the technology for extended periods, facilitating a more seamless integration of MAR into telemedicine and clinical workflows. This ease of use further promotes patient empowerment, enabling them to take an active role in understanding their own health data.

Prior literature has reported that 3D models have made telemedicine visits as effective as in-person visits [15]. This finding could be applied to MAR counseling, as the counseling process can be conducted through smartphones or tablet devices. The integration of MAR into telemedicine can potentially enhance the quality of counseling in the future.

### Limitations

However, the implementation of MAR consultation also has hidden challenges. Compared with traditional methods, MAR counseling demands a longer preparation time, additional training for clinical staff, and reliance on stable hardware and network infrastructure. Although MAR technology offers the advantage of accessing virtual resources anytime and anywhere, whether in medical education or clinical settings, its effectiveness and usability remain closely tied to technical settings. When users operate MAR systems in environments with limited bandwidth or outdated hardware, they may experience delays, failed file loading, or degraded 3D rendering performance. These limitations may result in reduced acceptance or satisfaction, regardless of the perceived educational or clinical value of the MAR content.

Additionally, the questionnaires used in this study were developed to reflect the unique attributes of MAR, which may limit direct comparison with SDM outcome measures (eg, the 9-item Shared Decision Making Questionnaire or the OPTION [Observing Patient Involvement] scale). Future studies should incorporate validated tools alongside MAR-specific items to improve generalizability and construct validity. Finally, the study was conducted at a single institution with a modest sample size, which may limit the broader applicability of our findings. Although the sample size was statistically adequate based on pilot data, larger multicenter trials are warranted to improve generalizability and explore subgroup effects. Future multicenter studies with larger and more diverse populations, time-matched conditions, and validated instruments are warranted to confirm the impact and feasibility of MAR counseling in thoracic surgery and beyond.

### Conclusions

MAR counseling demonstrated a significantly higher impact on SDM compared with traditional counseling for patients undergoing thoracic surgery. This indicates that MAR consultation is effective in supporting patients’ engagement in the decision-making process and enhancing their understanding, participation, and trust. Importantly, MAR counseling demonstrated statistically significant improvements in participants’ perceptions compared to traditional counseling. In contrast, under traditional counseling, patients often find it difficult to effectively organize their questions. Insufficient interaction with physicians may limit patients’ active participation in the SDM process. MAR consultation scored significantly higher in the same topics, better supported patients, and promoted communication between physicians and patients. However, we recommend conducting a multicenter study with a usability score assessment to validate the generalizability and feasibility of MAR counseling in future research. This will help ensure the widespread applicability and practicality of this approach in a broader context.

## Data Availability

The datasets generated and analyzed during this study are not publicly available due to institutional restrictions but are available from the corresponding author upon reasonable request.

## Appendix

Multimedia Appendix 1 Demonstration of both traditional and mobile augmented reality counseling methods (MP4 format).

Multimedia Appendix 2 Step-by-step demonstration of the mobile augmented reality 3D model creation and deployment (MP4 format).

Multimedia Appendix 3 CONSORT checklist.

## Abbreviations

AR: augmented reality
VR: virtual reality
MAR: mobile augmented reality
SDM: shared decision-making
